# Modifying a validated instrument: the Aberdeen varicose vein questionnaire

**DOI:** 10.1186/1745-6215-16-S2-P56

**Published:** 2015-11-16

**Authors:** Aleksandra Staniszewska, Seonaidh Cotton, Graeme MacLennan, Julie Brittenden

**Affiliations:** 1 University of Aberdeen, Aberdeen, UK

## 

The Aberdeen Varicose Vein Questionnaire (AVVQ) is validated and frequently used as a patient-reported disease-specific quality of life (QoL) outcome in trials of varicose veins. The AVVQ comprises a manikin diagram where patients draw their varicose veins and 12 questions including pain, skin changes, use of support stockings, appearance and impact. AVVQ scores range from 0 to 100 (worst possible QoL), with the manikin diagram contributing up to 22 points, depending on the extent of the varicose veins.

Our experience of using the AVVQ in the NIHR HTA funded CLASS trial comparing varicose vein treatments suggested that (i) a proportion of patients fail to complete the manikin diagram; (ii) scoring the manikin diagram was time-consuming and (iii) it was not possible to directly capture AVVQ responses from participants electronically (there was a need for a paper questionnaire). We therefore investigated the potential to use the AVVQ without the manikin diagram.

Using retrospective data from CLASS, we correlated the AVVQ (scored with and without the manikin diagram) against two clinical measures of disease severity. Individuals with more severe disease had higher AVVQ scores (regardless of whether the manikin diagram component was included or not).

In a prospective study of test-retest reliability involving 32 patients with varicose veins, we demonstrated good level of agreement between paired diagrams. In the same study, there was high level of agreement between two independent scorers.

Our results suggest that the AVVQ could be used without the manikin diagram, which could result in cost/time savings for trials.

